# Early stage breast cancer follow-up in real-world clinical practice: the added value of cell free circulating tumor DNA

**DOI:** 10.1007/s00432-022-03990-7

**Published:** 2022-04-09

**Authors:** E. La Rocca, M. C. De Santis, M. Silvestri, E. Ortolan, M. Valenti, S. Folli, F. G. de Braud, G. V. Bianchi, G. P. Scaperrotta, G. Apolone, M. G. Daidone, V. Cappelletti, G. Pruneri, S. Di Cosimo

**Affiliations:** 1 Breast Unit, Fondazione IRCCS Istituto Nazionale Dei Tumori, Milan, Italy; 2 Radiation Oncology, Fondazione IRCCS Istituto Nazionale Dei Tumori, Milan, Italy; 3 Biomarkers Unit, Department of Applied Research and Technological Development, Fondazione IRCCS Istituto Nazionale Dei Tumori, Milan, Italy; 4 Breast Cancer Surgery, Fondazione IRCCS Istituto Nazionale Dei Tumori, Milan, Italy; 5 Division of Medical Oncology, Fondazione IRCCS Istituto Nazionale Dei Tumori, Milan, Italy; 6 School of Medicine, University of Milan, Milan, Italy; 7 Radiology Unit, Fondazione IRCCS Istituto Nazionale Dei Tumori, Milan, Italy; 8 Scientific Directorate, Fondazione IRCCS Istituto Nazionale Dei Tumori, Milan, Italy; 9 Department of Pathology, Fondazione IRCCS Istituto Nazionale Dei Tumori, Milan, Italy

**Keywords:** Circulating tumor DNA, Follow-up, Breast cancer, Imaging

## Abstract

**Purpose:**

Physical examinations and annual mammography (minimal follow-up) are as effective as laboratory/imaging tests (intensive follow-up) in detecting breast cancer (BC) recurrence. This statement is now challenged by the availability of new diagnostic tools for asymptomatic cases. Herein, we analyzed current practices and circulating tumor DNA (ctDNA) in monitoring high-risk BC patients treated with curative intent in a comprehensive cancer center.

**Patients and methods:**

Forty-two consecutive triple negative BC patients undergoing neoadjuvant therapy and surgery were prospectively enrolled. Data from plasma samples and surveillance procedures were analyzed to report the diagnostic pattern of relapsed cases, i.e., by symptoms, follow-up procedures and ctDNA.

**Results:**

Besides minimal follow-up, 97% and 79% of patients had at least 1 non-recommended imaging and laboratory tests for surveillance purposes. During a median follow-up of 5.1(IQR, 4.1–5.9) years, 13 events occurred (1 contralateral BC, 1 loco-regional recurrence, 10 metastases, and 1 death). Five recurrent cases were diagnosed by intensive follow-up, 5 by symptoms, and 2 incidentally. ctDNA antedated disseminated disease in all evaluable cases excepted two with bone-only and single liver metastases. The mean time from ctDNA detection to suspicious findings at follow-up imaging was 3.81(SD, 2.68), and to definitive recurrence diagnosis 8(SD, 2.98) months. ctDNA was undetectable in the absence of disease and in two suspected cases not subsequently confirmed.

**Conclusions:**

Some relapses are still symptomatic despite the extensive use of intensive follow-up. ctDNA is a specific test, sensitive enough to detect recurrence before other methods, suitable for clarifying equivocal imaging, and exploitable for salvage therapy in asymptomatic BC survivors.

**Supplementary Information:**

The online version contains supplementary material available at 10.1007/s00432-022-03990-7.

## Introduction

Care procedures after curative treatment for primary breast cancer are common practice in clinical oncology. According to national and international guidelines (Runowicz et al. 2016; Cardoso et al. 2019; Barni et al. 2011), breast cancer follow-up should include regular updated history, physical examination (every third or sixth month), and annual mammography to focus on treatment related complications and cancer surveillance. Specifically, the purpose of mammographic follow-up is to detect local recurrences after breast-conserving surgery, which occur in up to 4% of early stage breast cancer cases (Yang et al. 2008), and to perform surveillance for contralateral breast cancer. Both local-/regional recurrences and contralateral cancer are amenable of treatment with curative intent. By contrast, intensive follow-up with the use of laboratory tests and imaging services, including bone scan, liver ultrasound, and chest radiograph, to screen asymptomatic breast cancer survivors for distant recurrence is discouraged as it failed to demonstrate any survival improvement (The GIVIO Investigators 1994; Palli et al. 1586; Rosselli Del Turco et al. 1994; Moschetti et al. 2016).

In the last decade, however, owing to the progress in imaging and availability of new effective therapies, secondary metastatic breast cancer has begun to be no longer considered as a fatal condition. In particular, patients with a limited number or sites of metastases are at an intermediate stage of tumor spread with limited metastatic potential (Weichselbaum and Hellman 2011 Jun), and can still be cured if treated with a multidisciplinary approach (Pagani et al. 2010). In addition, most patients with metastases are candidates for biological treatments including HER2, CDK and immune check point inhibitors that have significantly increased survival (Loibl et al. 2021). Based on these facts, detection of a low metastatic burden could lead to successful curative and/or chronic treatment. Therefore, despite the lack of evidence that active surveillance for metastatic disease improves outcomes in breast cancer survivors, intensive follow-up aimed at identifying metastases by the same imaging used for advanced disease has become quite common in clinical practice (Hahn et al. 2013; Natoli et al. 2014).

Meanwhile, an increased knowledge of breast cancer biology and the use of biotechnology in everyday diagnostics have enabled the development of biomarkers for detecting disseminated disease at a subclinical stage.

Circulating tumor DNA (ctDNA) is the fraction of cell-free circulating DNA that is derived from a patient's cancer. Breast cancer cells shed DNA into the blood, and interest in using ctDNA as a sensor to anticipate relapse in advance of clinical manifestation has grown in parallel with the improvement of techniques for its detection (Merker et al. 2018; Page et al. 2021). The present work builds on our experience with prospective longitudinal ctDNA monitoring before, during and after neoadjuvant therapy for triple negative breast cancer (Ortolan et al. 2021). Clinicians were blinded to the results of ctDNA assessment and continued to monitor patients according to their clinical practice. Here we report the surveillance procedures used in the sample of high-risk early stage breast cancer patients included in the previous study on blood-based genomics (Ortolan et al. 2021), and the numbers of recurrent disease diagnoses discovered based on symptoms or triggered by follow-up examinations, ctDNA or both in asymptomatic cases.

## Materials and methods

### Study cohort

Details on ctDNA analysis and predictive/prognostic significance during neoadjuvant therapy have been previously reported (Ortolan et al. 2021). All clinical and research staff were blinded as to ctDNA and follow-up outcomes, respectively. Eligible cases for the current analysis included consecutive patients with primary tumor lacking both hormone receptor and HER2-overexpression treated with curative intent; serial blood sampling (> 1 blood drawn) for ctDNA analysis; and detailed information on post-treatment procedures up to first relapse for a minimum of 1 year, or until disease recurrence, death for any cause or consent withdrawal, were collected as of September 30, 2021. Approval for this study was granted by the Institutional Ethical Committee, N.INT 196/14 and N. INT 199/15.

### Data sources

Data were obtained from electronic medical records. Variables of interest included primary tumor and patient characteristics, type of neoadjuvant therapy and surgical procedures (breast conserving surgery or mastectomy), type of medical consultations (oncological, gynecological, surgical), as well as diagnostic procedures and ctDNA occurrences in the absence of signs or symptoms suggestive of recurrence. Imaging was classified as recommended, *i.e.*, annual breast mammography, and non-recommended including breast ultrasound (US) and magnetic resonance imaging (MRI), chest and/or abdominal computed tomography (CT) or MRI, abdominal US, chest radiograph, radionuclide bone and ^18^F-FDG positron emission tomography(PET)/CT scan. To avoid capturing medical procedures performed as part of the diagnostic workup or possibly related to neoadjuvant therapy complications during active treatment, we designated the initiation of post-treatment care starting from the date of surgery.

### Statistical analysis

Data were analyzed using descriptive statistics. Means, ranges, and percentages were used to summarize patient clinical characteristics and visits. Proportions and frequencies were generated for recommended and non-recommended imaging services. Time to receipt of annual mammography, medical visits, blood examination and additional imaging for patients in each time-interval was estimated considering the date of surgery as starting point. Results from the evaluation of imaging services and their distribution for age and tumor stage were reported using histogram and box-plot. The association between ctDNA status during follow-up procedures within each patient were detailed using a heatmap representation. All descriptive analyses were performed using R software (version 4.1.1).

## Results

The study sample included 33 consecutive breast cancer survivors, after excluding 4 cases refusing consent to serial blood draws, 3 cases monitored in another hospital, and 2 cases with incomplete information on follow-up procedures. All included cases were treated with neoadjuvant chemotherapy for triple negative breast cancer and underwent surgery with curative intent between June 2013 and June 2018. Patient, primary tumor characteristics and main clinical outcomes are reported in Table 1. Median age was 44 (range 29–75) years. At diagnosis primary tumor size was > 2 and ≤ 5 cm (cT2) in 21/33 (63.3%), and > 5 cm (cT3) in 7/33 (21.2%) patients; clinical nodal status was positive (cN1–3) in 23/33 (69.7%) cases. No case of stage I was enrolled. Grade 3 was reported in most evaluable patients, and median Ki67 value was 70% (range 20%–90%). Two thirds of patients were treated with mastectomy after anthracycline and taxane-based neoadjuvant therapy. Pathological findings were available for all patients: 12 of 33 (36.4%) achieved a less than 1 cm residual disease response and additional 6 (18.2%) a pathological complete response, defined by absence of invasive cancer in breast and axillary nodes. With a median follow-up of 5.1 years (IQR 4.1–5.9), a total of 13 breast cancer events occurred, including 10 cases of secondary metastatic disease, 1 loco-regional recurrence, 1 controlateral breast cancer, and 1 death.

**Table 1:** Patient and primary tumor characteristics (*n* = 33) and type of breast cancer event (*n* = 13)

Characteristic	*n* (%)
Age	
< 50 years	22 (66.7%)
≥ 50 years	11 (33.3%)
Clinical tumor size	
cT1	2 (6.1%)
cT2	21 (63.6%)
cT3	7 (21.2%)
cT4	3 (9.1%)
Clinical Nodal Status	
cN0	10 (30.3%)
cN1–3	23 (69.7%)
Tumor Grade	
G2	1 (3.5%)
G3	28 (96.5%)
Subtotal	29
Missing	4 (12.1%)
Ki67	
< 50%	8 (26.7%)
≥ 50%	22 (73.3%)
Subtotal	30
Missing	3 (9.0%)
Neoadjuvant chemotherapy	
Anthracycline + taxane-based	27 (81.8%)
Platinum-based	4 (12.1%)
Anthracycline-based	2 (6.1%)
Type of surgery	
Conservative	11 (33.3%)
Mastectomy	22 (66.7%)
Pathological findings	
ypT_0_yN_0_ (pathological complete response)	6 (18.2%)
ypT_1_yN_0_	12 (36.4%)
ypT_1-3_yN_x-2_	13 (39.4%)
Breast cancer events (*n* = 13)	
Loco-regional relapse	1 (7.7%)
Contralateral breast cancer	1 (7.7%)
Distant metastases	10 (76.9%)
Death for any cause	1 (7.7%)

For the sake of completeness, patient and primary tumor characteristics of excluded cases (*n* = 9) are shown in Supplementary Table 1. Although the numbers are small, it appears that non participants were older, and diagnosed with less advanced and less aggressive primary tumors. Accordingly the number of events was low (*n* = 2). These favorable prognostic features might at least partially explain a low motivation to participate in a clinical and biological follow-up study.

### Recommended breast cancer follow-up procedures

Minimal follow-up was applied to most. Specifically, 97% and 85% of cases had at least one medical examination and annual mammography, respectively (Table 2). Notably, 100% of patients undergoing breast conserving surgery received annual mammography regardless of the time from surgery (data not shown). The distribution of patients receiving the recommended minimal follow-up remained high even years after surgery.

**Table 2:** Use of minimal and non-recommended follow-up in the study cohort overall and according to the time from surgery

Time interval	Patients in the time interval	Mammograms	Medical visits	Laboratory tests	Additional imaging
Overall	33	28 (85%)	32 (97%)	26 (79%)	32 (97%)
Years after surgery					
1	30	23 (77%)	19 (63%)	12 (40%)	24 (80%)
2	25	21 (84%)	18 (72%)	12 (48%)	23 (92%)
3	21	19 (90%)	17 (81%)	9 (43%)	17 (81%)
4	20	18 (90%)	15 (75%)	10 (50%)	14 (70%)
5	18	14 (78%)	8 (44%)	5 (28%)	8 (44%)

### Additional follow-up procedures

**Fig. 1:**
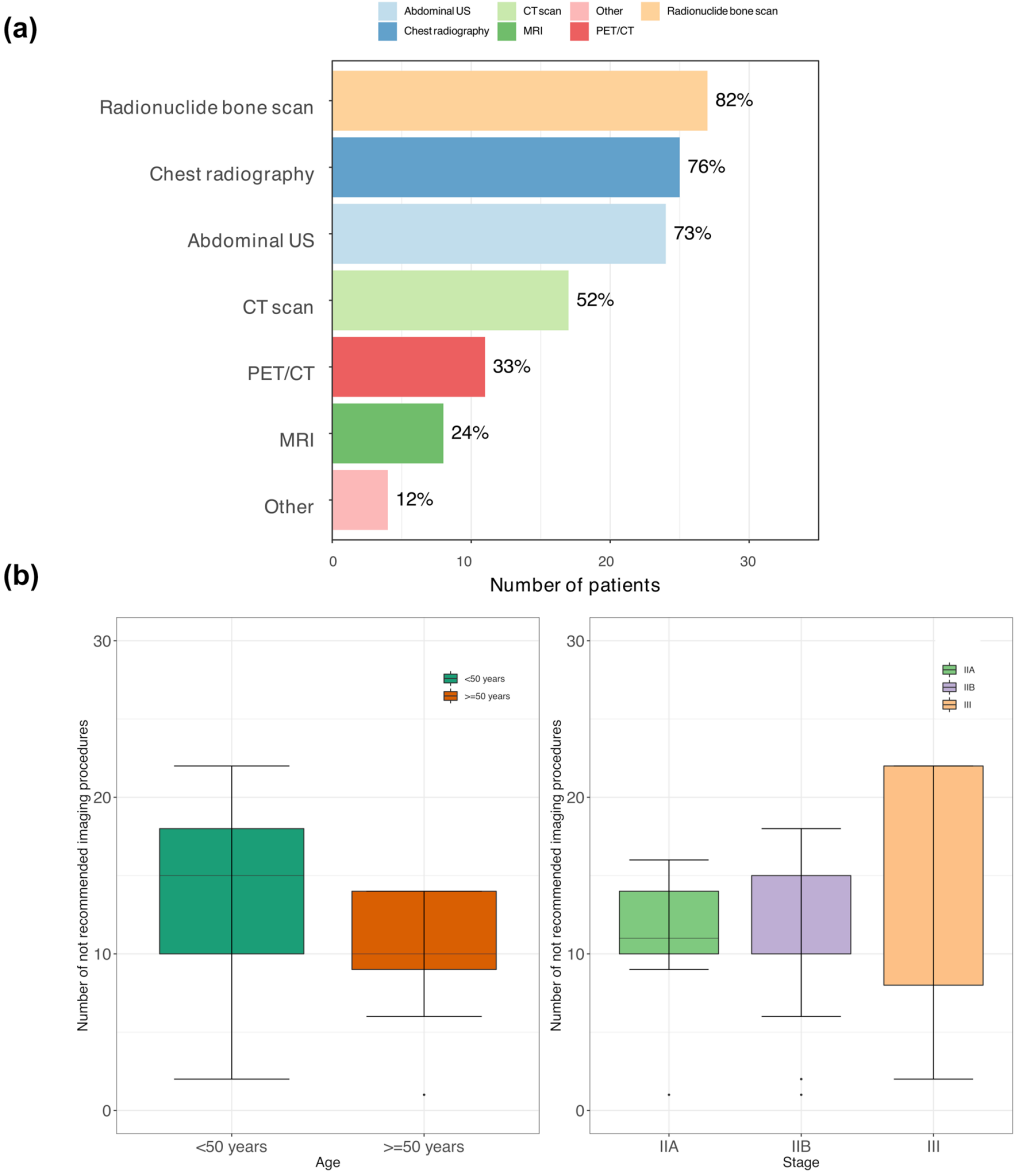
**a** Histograms show the number and percentage of non-recommended imaging services by type; **b** Box plots report the number of non-recommended imaging services according to patient age and tumor stage

Ninety-seven and 76% of sample had at least 1 or 2 non-recommended imaging examinations, respectively. The most common were bone scan (82%), chest radiograph (76%), and abdominal US (73%) (Fig. 1, panel a). Notably one third of sample received at least one CT or PET/CT scan. Neither age nor stage were associated with the use of additional follow-up procedures (Fig. 1, panel b). Not recommended procedures were distributed uniformly according to the time from surgery, suggesting that perception of risk was not limited to the first years after treatment with curative intent (Table 2).

### Suspicious findings at follow-up procedures

During the study period, a total of 7 patients had suspicious findings at surveillance procedures. Five of these patients were finally diagnosed with recurrent breast cancer. Notably, ctDNA detection antedated suspected lesions of on average 3.81 months [standard deviation (SD) = 2.68], and the final diagnosis of recurrence of 8 (SD = 2.98) months. Of note, 2 patients with suspected lesions not confirmed by subsequent additional examinations had undetectable levels of ctDNA.

### Detection of recurrence

**Fig. 2:**
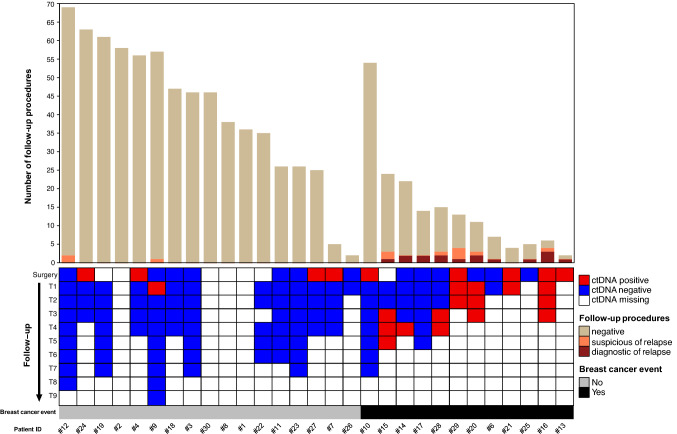
Bar plot reports the number of follow-up procedures for each patient as negative (pale brown), suspected for malignancy (orange), or diagnostic of recurrence (bordeaux). The heatmap columns report the results of ctDNA assessment for each patient of the study cohort before surgery and at intervals of 4–6 months (*T*_1_, *T*_2_, *T*_*n*_) during follow-up as detectable (red), undetectable (blue), or missing (white). Breast cancer event occurrence (black) or absence (grey) is reported as a bar on the bottom of the figure. To be noted: patient #10 had a second primary breast cancer at prophylactic contralateral mastectomy; patient #21 died for causes unrelated to cancer and its treatment; patients #1, #2, #8 and #30 lack ctDNA information due to wild type primary tumors; patients #5 and #31–33 are not reported due to non-evaluable primary tumor mutational profile

Figure 2 shows intensive follow-up procedures and ctDNA details in recurrent and non recurrent cases. Aside 5 recurrent cases diagnosed by scheduled follow-up (#5, 14, 15, 16, 17); the remainder presented symptomatically (#6, 20, 25, 28, 29) or incidentally on procedures carried out for concurrent medical condition (#10 and #13).

ctDNA remained detectable despite treatment with curative intent in three patients (#13, #16, #29) all experiencing breast cancer recurrence within the first year from surgery (specifically, 9.1, 11.2 and 9.1 months). ctDNA turned positive after surgery in three other cases (#15, #20, #28) anticipating overt metastases by 13, 6.3, and 7 months, respectively. ctDNA failed to anticipate the diagnosis of secondary primary (#10) and loco-regional recurrence (#6), bone (#17) and liver only metastatic disease (#14). Notably, in the latter case the ctDNA became detectable at the time of diagnosis by imaging.

All cases without any evidence of recurrence had undetectable levels of ctDNA at the time of data lock.

## Discussion

To date there has been no study for breast cancer follow-up attempting to evaluate the value of ctDNA in the framework of real world intensive follow-up. In the current study including consecutive triple negative breast cancer patients treated and monitored for recurrence in a comprehensive cancer center, it is shown that intensive follow-up procedures represent a common practice but still miss many recurrent cases. Beside, ctDNA assessment in serial plasma samples holds the potential to antedate the diagnosis of recurrence in a subclinical phase and to fix equivocal suspected findings at follow-up procedures.

The expanding options for systemic treatment of breast cancer patients suggest that proactive research into recurrent disease is worthwhile. It is a common belief that diseases are more treatable when diagnosed at an early rather than advanced stage, and this applies also to metastatic breast cancer (Llombart-Cussac et al. 2014; Cuyún Carter et al. 2021). Moreover, breast cancer encompasses a spectrum extending from localized to systemic disease with many intermediate states including oligometastases, which are characterized by minimal spreading potential (Weichselbaum and Hellman 2011). Finally, the diagnosis of recurrence in asymptomatic cases avoids the threat of visceral crisis, defined as signs, symptoms, and laboratory work-up of organ dysfunction and rapid disease progression precluding the use of most available therapies (Cardoso et al. 2020). Hence, it is not surprising that experienced physicians from the multidisciplinary breast cancer unit of our Institution ordered additional services over those recommended by national and international guidelines in up to 97% of cases. The data confirm previous studies showing that recommended follow-up procedures are prescribed by a minority (10–45%) of medical oncologists (Keating et al. 2007; Adesoye et al. 2018; Matro and Goldstein 2014). By contrast, young age and advanced stage at initial diagnosis were not confirmed as being associated with non-recommended imaging (Hahn et al. 2013; Natoli et al. 2014; Rabin et al. 2014). This finding is probably due to the homogeneity of our study population, consisting of patients with a median age of 43 years with stage II and III triple negative breast cancer.

An optimal follow-up program should identify recurrent cases at such an early stage that treatment might eliminate minimal disease in a manner similar to adjuvant therapy in patients with high-risk primary breast cancer (Hortobagyi 2002). This represents a crucial need which emerged early, despite remaining unmet for a long time due to the lack of accurate diagnostic tools (Cock et al. 2021).

Our study shows that ctDNA identifies 75% of evaluable cases developing secondary metastatic breast cancer in advance of clinical, laboratory and instrumental diagnosis of up to 13 months. Whether an advance of this magnitude is sufficient for curative treatment needs to be investigated in prospective studies. ctDNA appears to be more sensitive for detection of disseminated disease than loco-regional recurrence and contralateral breast cancer, confirming previous studies (Cosimo et al. 2019; Garcia-Murillas et al. 2019; Coombes et al. 2019). Persistence or elevation of ctDNA levels always lead to the diagnosis of recurrent disease, suggesting that this tool is specific to the breast cancer condition. In addition, an interesting but still unconfirmed result, is that the absence of ctDNA in suspected cases could increase the specificity of imaging used in intensive follow-up and avoid further unnecessary investigations.

Feasibility of ctDNA analysis should be considered when discussing the *pros* and *cons* of this approach. Tracking ctDNA is challenging from a diagnostic perspective, as cancer recurrence happens in the future, and no current standard metric exists against which to measure a true positive or negative test. However, we followed-up patients for a sufficiently long period, considering that triple negative breast cancer recurrences are expected within the first 3 years from surgery (Colleoni et al. 2016). Furthermore, at the time of diagnosis of overt metastases, ctDNA was detectable in all but two cases with single site metastases. These results indicate that almost all secondary metastatic breast cancer is associated with increased ctDNA, at least in triple negative breast cancer, and are in line with previously reported studies showing that relapse without ctDNA detection before or at the time of relapse occurs in less than 20% of cases (Garcia-Murillas et al. 2019; Coombes et al. 2019).

In addition to feasibility, the potential emotional impact of using ctDNA in the follow-up of patients with early stage breast cancer should be considered. Having serial assessment of ctDNA may give a feeling of security to the patient, but it may also cause a feeling of anxiety (Thewes et al. 2012; Mehnert et al. 2009). Consistent with this, we noted that of the initial cohort identified as suitable for the study, informant consent was withheld in 4 cases. Patient counseling about the advantages and disadvantages of ctDNA measurement is essential.

Some limitations of our study should be noted. First, it is a single-center retrospective study. Although our experience, similarly to others (Garcia-Murillas et al. 2019; Coombes et al. 2019), showed that ctDNA tracking by digital droplet PCR achieves reliable detection of 0.01% from 20 ng DNA, our detection capability was limited to the evaluation of only one or at most two mutations identified from patient tumor biopsies. We reason that future efforts will require by large gene panel to identify more mutations to track in all patients (Ignatiadis et al. 2021). In addition, volumes of plasma processed were small, meaning cell free DNA yield and the number of genomic equivalents sampled was low.

Despite these limitations, in a population undergoing serial measurements of ctDNA in the follow-up of triple negative breast cancer patients treated with neoadjuvant therapy, persistence or re-appearance of ctDNA is the first sign of secondary metastatic breast cancer irrespective of other signs or symptoms in about three quarters of patients with disseminated disease. These results advocate an integration of ctDNA analysis to identify recurrences earlier than intensive follow-up, to assist in the diagnostic procedures of suspicious findings, and to develop salvage adjuvant therapeutic strategies.

## Supplementary Information

Below is the link to the electronic supplementary material.

Supplementary file1 (DOCX 16 KB)

## Data Availability

Original data are available upon reasonable request to the corresponding author.
