# Supplementary material for: Early stage breast cancer follow-up in real-world clinical practice: the added value of cell free circulating tumor DNA
Source: J Cancer Res Clin Oncol. 2022 Apr 9;148(6):1543–50. doi: 10.1007/s00432-022-03990-7 (PMC9114063; doi:10.1007/s00432-022-03990-7)
Supplement: Supplementary file 1 — Supplementary file1 (DOCX 16 KB) [file 432_2022_3990_MOESM1_ESM.docx]

**Supplementary Table 1.** Patient and primary tumor characteristics of excluded cases (n=9) and type of breast cancer event (n=2)

| Characteristic | N (%) |
| --- | --- |
| Age |  |
| < 50 years | 5 (55.5%) |
| ≥ 50 years | 4 (44.5%) |
| Clinical Tumor Size |  |
| cT1 | 0 |
| cT2 | 8 (88.9%) |
| cT3 | 1 (11.1%) |
| cT4 | 0 |
| Clinical Nodal Status |  |
| cN0 | 5 (55.5%) |
| cN1-3 | 4 (44.5%) |
| Tumor Grade |  |
| G2 | 0 |
| G3 | 9 (100%) |
| Ki67 |  |
| < 50% | 3 (37.5%) |
| ≥ 50%  Subtotal | 5 (62.5%)  8 |
| *Missing* | 1 (11.1%) |
| Neoadjuvant chemotherapy |  |
| Anthracycline + taxane-based  Platinum-based  Other | 6 (66.7%)  2 (22.2%)  1 (11.1%) |
| Type of surgery |  |
| Conservative | 6 (66.7%) |
| Mastectomy | 3 (33.3%) |
| Pathological Findings |  |
| ypT_0_yN_0_ (pathological complete response) | 0 |
| ypT_1_yN_0_ | 4 (44.5%) |
| ypT_1-3_yN_x-2_ | 5 (55.5%) |
| Breast cancer events (n=2) |  |
| Distant metastases | 2 (100%) |
